# Changes in Heart Rate and Heart Rate Variability During Surgical Stages to Completed Fontan Circulation

**DOI:** 10.1007/s00246-021-02595-0

**Published:** 2021-04-10

**Authors:** Jenny Alenius Dahlqvist, Urban Wiklund, Marcus Karlsson, Katarina Hanséus, Eva Strömvall Larsson, Jens Johansson Ramgren, Håkan Berggren, Annika Rydberg

**Affiliations:** 1 Department of Clinical Sciences, Pediatrics, Umeå University, 90185 Umeå, Sweden; 2 Department of Radiation Sciences, Biomedical Engineering, Umeå University, Umeå, Sweden; 3 Department of Clinical Sciences Lund, Children Heart Centre, Skåne University Hospital Lund, Lund, Sweden; 4 Department of Cardiology, Institute of Clinical Sciences, The Queen Silvia Children’s Hospital, Sahlgrenska University Hospital, Gothenburg University, Gothenburg, Sweden; 5 Department of Pediatric Cardiac Surgery, Children’s Heart Center, Skåne University Hospital Lund, Lund, Sweden; 6 Department of Pediatric Cardiac Surgery, Children’s Heart Center, The Queen Silvia Children’s Hospital, Göteborg, Sweden

**Keywords:** Fontan circulation, Bidirectional Glenn procedure, Arrhythmia, Heart rate variability, Total cavopulmonary connection, Univentricular heart defect

## Abstract

Arrhythmia is related to heart rate variability (HRV), which reflects the autonomic nervous regulation of the heart. We hypothesized that autonomic nervous ganglia, located at the junction of the superior vena cava’s entrance to the heart, may be affected during the bidirectional Glenn procedure (BDG), resulting in reduced HRV. We aimed to investigate changes in heart rate and HRV in a cohort of children with univentricular heart defects, undergoing stepwise surgery towards total cavopulmonary connection (TCPC), and compare these results with healthy controls. Twenty four hours Holter-ECG recordings were obtained before BDG (*n* = 47), after BDG (*n* = 47), and after total cavopulmonary connection (TCPC) (*n* = 45) in patients and in 38 healthy controls. HRV was analyzed by spectral and Poincaré methods. Age-related *z* scores were calculated and compared using linear mixed effects modeling. Total HRV was significantly lower in patients before BDG when compared to healthy controls. The mean heart rate was significantly reduced in patients after BDG compared to before BDG. Compared to healthy controls, patients operated with BDG had significantly reduced heart rate and reduced total HRV. Patients with TCPC showed reduced heart rate and HRV compared with healthy controls. In patients after TCPC, total HRV was decreased compared to before TCPC. Heart rate was reduced after BDG procedure, and further reductions of HRV were seen post-TCPC. Our results indicate that autonomic regulation of cardiac rhythm is affected both after BDG and again after TCPC. This may be reflected as, and contribute to, postoperative arrhythmic events.

## Introduction

Since the first description of the Fontan procedure [1], the surgical technique used for palliation of children with univentricular heart defects has evolved. Currently, the most common technique used is the staged Fontan surgery, in which the bidirectional Glenn procedure (BDG) is performed during the first year of life, followed by the total cavopulmonary connection (TCPC) at 2–4 years of age. In the majority of cases, neonatal surgery has been performed prior to BDG in order to ensure sufficient blood supply to the systemic and pulmonary circulation until the child is suitable for BDG. In parallel to development of surgical techniques, survival of children with univentricular heart defects has also improved. However, long-time morbidity and mortality is still substantial including arrhythmic events, a serious complication [2–5]. Sinus node dysfunction has been reported in 30–45% of patients with Fontan circulation [6, 7], with an increasing number with longer follow-up after Fontan.

Heart rate variability (HRV) measures the duration of consecutive RR intervals on an electrocardiogram (ECG) to assess beat-to-beat variation and is a well-established method of examining cardiac autonomic nervous activity. HRV increases with age during childhood; however, the increase is higher during infancy than late childhood [8].

Reduced HRV is associated with an increased risk of cardiovascular events in adult populations without known cardiovascular disease [9]. There is also evidence that reduced HRV is associated with an increased risk of sudden cardiac death in patients with congenital heart disease [10] and with cardiac arrest after cardiac surgery in neonates [11].

Earlier studies have shown reduced HRV in patients with Fontan circulation [12, 13]. In patients operated with two different methods of the TCPC, lateral tunnel, and extracardiac conduit, no significant HRV differences were found [7, 14]. However, little is known about the change in HRV of patients with univentricular heart defects during the progression of surgical steps towards complete Fontan circulation. Currently the only study following HRV in patients pre-BDG, post-BDG, and post-TCPC was carried out on short-term (15 min) ECG recordings; this study showed significantly reduced HRV after each surgery compared to follow-up at a later date [15].

Autonomic nervous ganglia density and distribution varies within the heart, with a moderately dense node located near the posterior surfaces of the superior vena cava (SVC) [16]. During the BDG procedure, preceding TCPC, the SVC is detached at the entrance to the heart and the cardiac end is oversewn. We hypothesized that parasympathetic ganglia may be affected during this process, resulting in a reduction of HRV. Therefore, our aim was to investigate changes in HRV in a cohort of children with univentricular heart defects, undergoing staged TCPC, and compare these results with healthy controls of the same age.

## Materials and Methods

The study included patients with univentricular heart defects operated with Fontan surgery at the two centers for pediatric thoracic surgery in Sweden, the University Hospitals in Lund and Gothenburg.

### Surgical Technique

The Fontan operation was performed in two steps, in most cases, after a palliative neonatal procedure. Firstly, the BDG procedure was performed via a median sternotomy on a beating heart by cannulation of the SVC, the right atrium, and aorta. The SVC was detached at the entrance to the heart and the cardiac end oversewn. The SVC and right pulmonary artery were then connected with an end-to-side anastomosis.

Secondly, TCPC was completed by creation of an intra-atrial tunnel or an extracardiac conduit. The intra-atrial tunnel surgery was performed via a median resternotomy, cardiopulmonary bypass and cardioplegic arrest (or induced ventricular fibrillation). A GORE-TEX® baffle was constructed to direct flow from inferior vena cava (IVC) to the superior end of an atriopulmonary anastomosis. The extracardiac conduit TCPC was performed via a median resternotomy. After atrial, bicaval and aortic cannulation, cardiopulmonary bypass was started. The IVC was transected and the cardiac end was oversewn. A GORE-TEX® tube graft was sutured to the IVC, and the other end connected to the right pulmonary artery. The procedure was performed on a “beating heart”.

### Data Collection

Ambulatory Holter-ECG recordings were performed in patients before BDG (pre-BDG), after BDG (post-BDG) and after TCPC (post-TCPC). Patients treated with beta-blockers or with a permanent pacemaker were excluded. Clinical records were reviewed for gender, anatomical diagnosis, surgical technique, age at time of each surgery, and complications such as protein losing enteropathy (PLE), heart transplantation or death. Records also provided information regarding saturation by pulse oximetry, medication, and echocardiographic findings at time points close to Holter-ECG assessment and procedures. Echocardiographic data included assessment of ventricular function and atrioventricular (AV) valve insufficiency. Semi-quantitatively assessed ventricular function was graded as “normal”, “mildly depressed”, “moderately depressed”, or “severely depressed” and the AV valve regurgitation was graded as “none”, “mild”, “moderate”, or “severe”.

### Controls

The healthy control group consisted of 38 children 24 female, (63%), and were recruited from the local population. All controls had normal baseline ECGs and echocardiographic scans. The median age was 20 (range 0–65) months.

### Ambulatory 24 h Holter-ECG and HRV Analysis

Heart rate variability analysis was carried out on five lead 24 h Holter-ECG traces collected during normal daily activity. One of the two channels (V2 or V5) was analyzed for arrhythmias before HRV analysis. Heartbeats were classified as: normal, supraventricular extra systolic, ventricular extra systolic, or beats of uncertain origin. All ECG data were reviewed and edited by one analyst who corrected undetected or misclassified heartbeats and excluded periods of noise or artifact.

Heart rate variability (HRV) was analysed using power spectrum analysis and Poincaré plots. Power spectrum analysis of the beat-to-beat fluctuations in RR intervals was performed by fast Fourier transformation as described previously [14]. Spectral power (*P*) was determined for three spectral components: *P*_VLF_; very low-frequency region [0.003–0.04 Hz (Hz)]; *P*_LF_; low-frequency region (0.04–0.15 Hz); and *P*_HF_; high-frequency region (above 0.15 Hz). HF represents mainly parasympathetic activity, whereas LF represents the combined sympathetic and parasympathetic effect on cardiac autonomic nervous modulation. The LF/HF ratio was also calculated. Finally, total power (*P*_tot_) was determined as the sum of the three spectral components. All spectral indices were calculated as average data over the complete recording period. Poincaré analysis is a geometric method in which each *RR* interval is plotted, on a scatter plot, as a function of the previous *RR* interval. HRV was quantified by the standard deviation (*SD*) in two perpendicular directions: *SD2*, the *SD* along the line of identity that represents the changes in mean *RR*; and *SD1*, the *SD* along the line that is perpendicular to the line of identity, representing the magnitude of the beat-to-beat variability in *RR* [17]. Recordings shorter than 10 h were excluded.

### Statistical Analysis

Data were presented as frequencies, percentages, or medians with ranges (minimum and maximum). All HRV variables were log-transformed due to their skewed distribution and presented as age-dependent *Z* scores based on control data. Linear regression modeling was used since nearly all HRV indices increased linearly between 0 and 9 years of age in controls. However, since LF/HF showed a U-shaped pattern over this age, a quadratic regression line was used. Thus, a *Z* score of 0 is equivalent to the mean for controls of the same age, and a *Z* score of 1 is equivalent to one *SD* above the mean of the controls. *Z* scores for the three different stages in patients (pre-BDG, post-BDG and post-TCPC) and controls were compared using linear mixed effects modeling. Differences between different stage recordings in patients and stages compared to controls Holter-ECG data were modeled using a nominal categorical variable with four levels. The linear mixed effects models also included random effects for each subject since each individual patient had 1–3 Holter recordings. Additionally, changes between the stage before BDG (pre-BDG) and stage after BDG (post-BDG), and after the TCPC operation (post-TCPC) were analysed using non-parametric Wilcoxon test, in which analysis only included patients which had one, or both, of these paired recordings. Statistical significance was defined as *p* < 0.05. All data and statistical analysis were performed using Matlab R2018b (Mathworks Inc, Natick, MN, USA) and IBM SPSS Statistics for Windows, Version 24.0 (IBM Corp. Armonk, NY, USA).

## Results

The study included 89 patients with univentricular heart defects (34% female). Thirty-three patients (37%) had hypoplastic left heart syndrome, 15 (17%) had tricuspid atresia, 11 (12%) had an unbalanced atrioventricular septal defect with hypoplasia of the left ventricle, 10 (12%) had double inlet left ventricle, 9 (10%) had complex double outlet right ventricle, 4 (4%) had critical aortic stenosis with hypoplasia of the left ventricle, 3 (3%) had a ventricular septal defect (2 cases with straddling of atrioventricular valves and 1 case of multiple ventricular septal defects with coarctation of aorta and left ventricular hypoplasia), 2 (2%) pulmonary atresia with intact ventricular septum, 1 (1%) aortic atresia and 1 (1%) Shones complex. Concerning the morphology of the systemic ventricle; 58 (65%) patients presented with right ventricular morphology, 28 (32%) with left ventricular morphology, and in 3 (3%) cases the patient had both left and right ventricle morphology present.

Neonatal surgery was performed in 83 of the patients: 44 (49%) had Norwood surgery [16 with a Blalock-Taussig (BT) shunt and 28 with a Sano-shunt], 24 (27%) received a shunt (modified BT or central shunt), 16 (18%) had a pulmonary artery banding and 13 (15%) were operated on with Damus-Kaye-Stansel anastomosis and shunt. BDG surgery was performed at median age of 5.9 (1.5–54.3) months. At the end of the study 71 of 89 patients had a complete Fontan circulation: 68 (96%) had an extracardiac TCPC and 3 (3%) had a lateral tunnel TCPC. Median age at TCPC was 2.6 (1.5–9.6) years. During follow-up 2 (2%) patients had a permanent pacemaker implantation, 1 (1%) patient developed protein losing enteropathy and 10 (11%) had received a heart transplant or died.

HRV was analyzed in pre-BDG Holter-ECGs in 47 patients (median age 4.5 months, range 0.6–53.4 months), post-BDG in 47 patients (median aged 25 months, range 7–40 months), post-TCPC in 45 patients, aged 3.7 years, range 2.2–8.8 years. Repeated Holter recordings were available for 39 patients; at all stages (*n* = 11), pre- and post-BDG (*n* = 8), pre-BDG and post-TCPC (*n* = 7), post-BDG and post-TCPC (*n* = 13). Twenty-one, 15 ,and 14 patients performed only one Holter: pre-BDG, post-BDG, and post-TCPC, respectively. A total of 139 recordings were analyzed. Patients were unavailable for Holter-ECG recordings for multiple reasons. In 18 cases, the final TCPC stage was not performed due to age (too young) (*n *= 7), operation not possible due to hemodynamic parameters or small size of the pulmonary arteries (*n* = 3), four had a heart transplantation, three died and one emigrated. During follow-up ten patients had a heart transplant or died (3 after TCPC).

See Table 1 for age at time of each procedure, time between surgeries and Holter-ECG registration, echocardiographic findings, medication and blood oxygen saturation.

**Table 1:** Clinical data at the time for Holter-ECG recordings

	Pre-Glenn (*n* = 47)	Pre-TCPC (*n* = 47)	Post- TCPC (*n* = 45)
Age: median (min–max)	5 (1–53) months	25 (7–40) months	3.7 (2.2–8.8) years
Time from last surgery, median (min–max), months	From stage 1 4 (0.5–53)	From BDG 16 (1–33)	From TCPC 9 (1–49)
^a^Ventricular function (echo) (*n*, valid %)			
Normal	37 (88)	30 (97)	24 (86)
Mildly depressed	5 (12)	1 (3)	4 (14)
Moderately depressed	0	0	0
Severely depressed	0	0	0
AV regurgitation (echo) (*n*, valid %)			
None	16 (38)	13 (42)	4 (14)
Mild	21 (50)	14 (45)	21 (72)
Moderate	4 (10)	3 (10)	4 (14)
Severe	1 (2)	1 (3)	0
Saturation %, median (range)	80 (68–90)	84 (73–92)	96 (92–99)
^b^Medication (*n*, valid %)			
Aspirin	23 (53)	12 (40)	17 (59)
Warfarin	2 (5)	2 (6)	8 (18)
Diuretics	27 (64)	21 (33)	16 (55)
ACE inhibitors	15 (36)	17 (26)	12 (41)
Beta-blockers	1 (2)	0	0
Digoxin	6 (14)	0	0

Stage 1—Neonatal surgery

*AV* Atrioventricular, *ASA* acetylic salicylic acid, *ACE* angiotensin converting enzyme, *PA* pulmonary artery

^a^Missing data echocardiographic findings in 5 patients Holter 1, 21 patients Holter 2, 17 Holter 3

^b^Missing data medication (no missing data concerning beta-blockers), saturation Holter 1: 5, Holter 2: 16, Holter 3: 16 patients

**Fig. 1:**
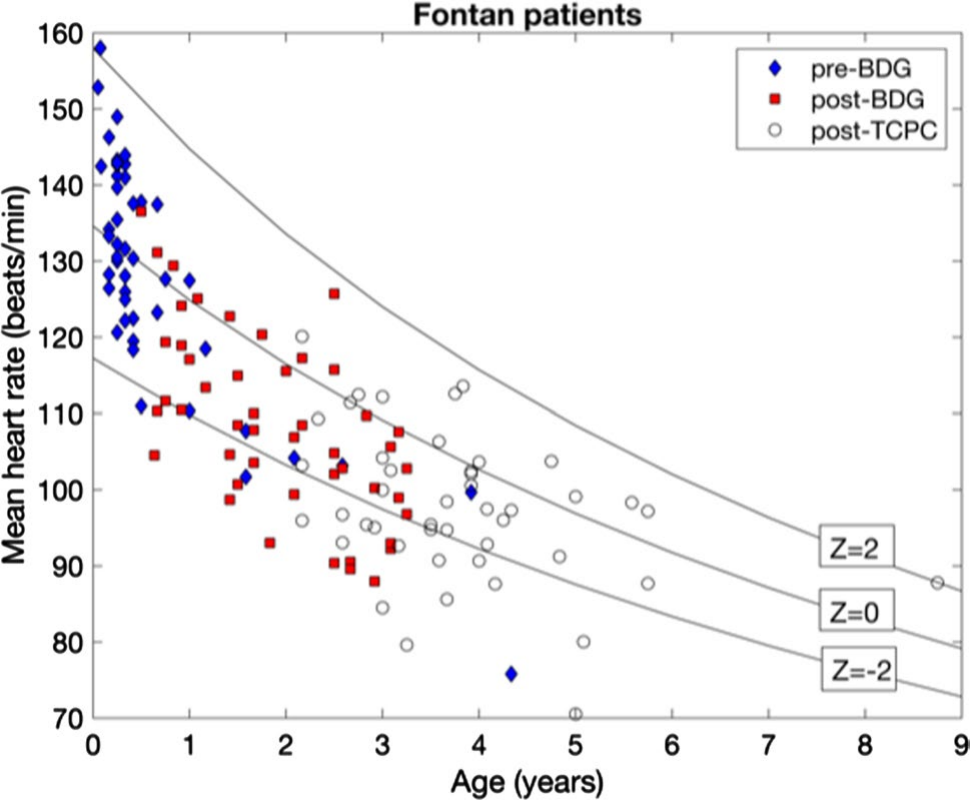
Mean heart rate before bidirectional Glenn surgery (BDG), (pre-BDG), after BDG/before TCPC (post-BDG), and after TCPC (post-TCPC). Solid lines show the estimated age dependency in control subjects (*Z* score = 0) and the corresponding 95% confidence intervals (*Z* score = ±2)

**Fig. 2:**
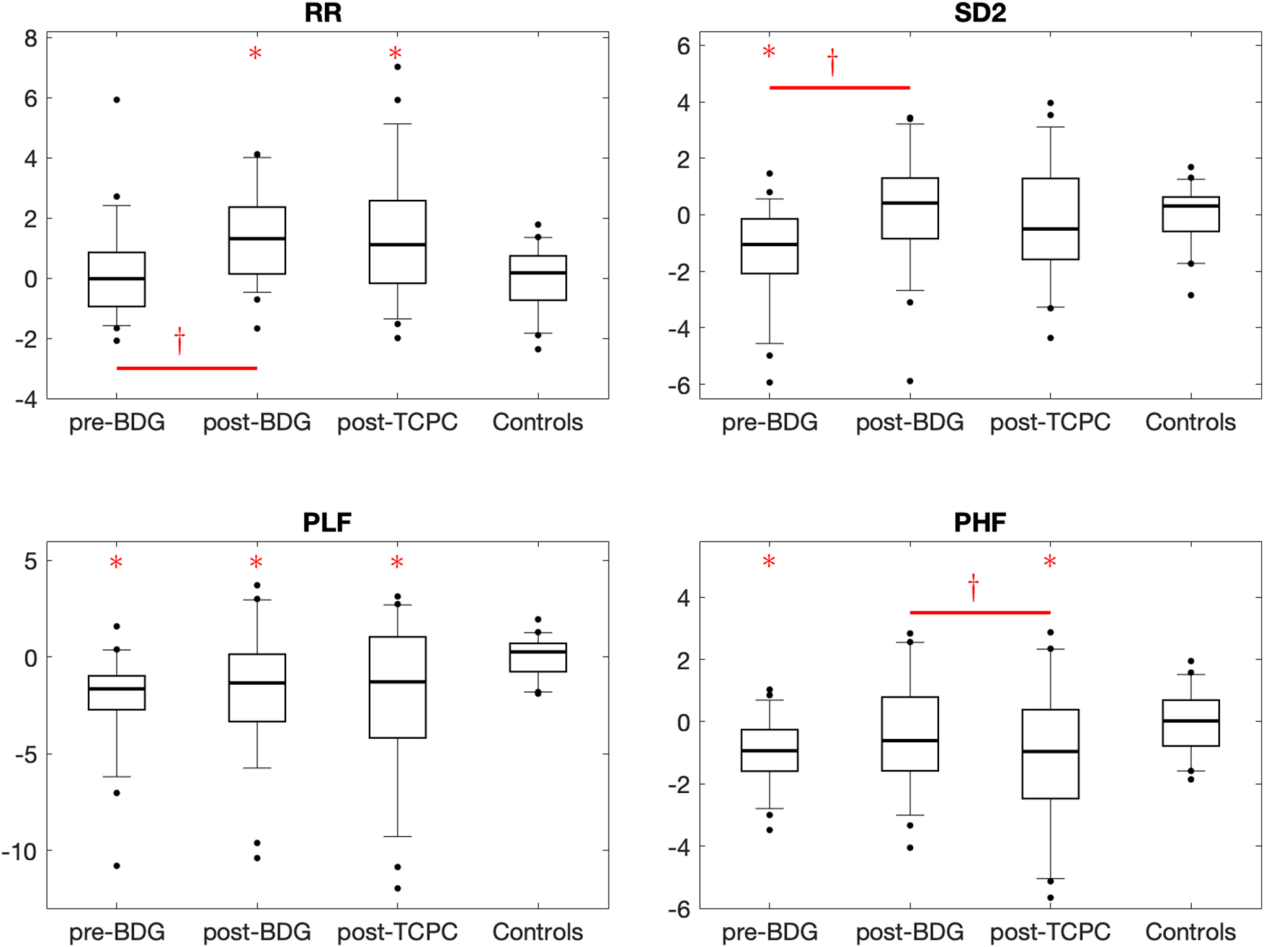
HRV measures expressed as *z* scores before bidirectional Glenn surgery (pre-BDG), after BDG/before TCPC (post-BDG), and after TCPC (post-TCPC). Boxes show median and interquartile range, whiskers show 90% percentiles. *p < 0.05 in comparison between patients and controls. *Ɨ* = *p* < 0.05 before and after BDG and before and after TCPC, respectively

In 6% (3/47) of the pre-BDG, 30% (14/47) of the post-BDG and 31% (14/45) of the post-TCPC recordings, patients showed marked sinus bradycardia (RR > 2 *Z* score) indicating sinus node dysfunction (Fig. 1). In pre-BDG Holter-ECGs 1/47; in post-BDG Holter-ECGs 4/47 and in post-TCPC Holter-ECGs 1/45 patients showed intermittent episodes of nodal rhythm. On comparison of HRV parameters via the mixed model analysis method, in which means of *Z* scores for HRV parameters [spectral power analysis (*P*_tot,_ *P*_VLF,_ *P*_LF,_ *P*_HF,_ *P*_LF_/*P*_HF_) and in Poincaré analysis *SD2* and *SD1*/*SD2* ratio] were compared, a significantly lower HRV was revealed in patients before BDG compared to healthy controls (Table 2, Fig. 2). The *RR* interval was significantly increased in patients after BDG compared to before BDG (Table 2, Fig. 2). Also, the *SD2* was significantly increased after BDG (Table 2, Fig. 2). Compared to healthy controls, the patients post-BDG had a significantly longer RR interval, decreased *P*_tot_ and *P*_LF_ (Table 2, Fig. 2). In patients after TCPC procedure, HRV parameters (*P*_tot,_ *P*_VLF,_ *P*_LF,_ *P*_HF,_ *P*_LF_/*P*_HF_) and in Poincaré analysis *SD2*_,_ and *SD1*/*SD2* ratio were decreased compared to before TCPC (post-BDG) (Table 2, Fig. 2). Compared with healthy controls, patients with TCPC showed longer RR intervals and reduced HRV in *P*_tot,_ *P*_VLF,_ *P*_LF,_ and *P*_HF,_ and in *SD1*_,_ and *SD1*/*SD2* ratio calculated via Poincaré analysis (Table 2, Fig. 2).

**Table 2:** Comparisons of *Z* scores for HRV at different Fontan stages

	PreBDG (1) (*n* = 47)	Post-BDG (2) (*n* = 47)	PostTCPC (3) (*n* = 45)	*p* value 1 vs 2	*p* value 2 vs 3	*p* value 1 vs C	*p* value 2 vs C	*p* value 3 vs C
*RR*	0.21 (0.21)	1.47 (0.22)	1.35 (0.22)	< 0.001	0.65	0.51	< 0.001	< 0.001
*P*_tot_	−1.40 (0.29)	−0.93 (0.29)	−1.46 (0.30)	0.20	0.14	0.002	0.04	0.002
*P*_VLF_	−1.48 (0.35)	−0.93 (0.35)	−1.15 (0.35)	0.22	0.63	0.006	0.08	0.03
*P*_LF_	−1.81 (0.36)	−1.42 (0.36)	−2.02 (0.37)	0.38	0.17	0.001	0.01	< 0.001
P_HF_	−0.78 (0.21)	−0.43 (0.21)	−1.36 (0.22)	0.17	< 0.001	0.02	0.19	< 0.001
*P*_LF_/*P*_HF_	−0.97 (0.22)	−0.44 (0.22)	0.36 (0.23)	0.08	0.01	0.004	0.19	0.28
*SD1*	0.01 (0.22)	−0.06 (0.22)	−1.05 (0.22)	0.81	< 0.001	0.97	0.87	0.002
*SD2*	−1.32 (0.23)	0.22 (0.23)	−0.14 (0.23)	< 0.001	0.19	< 0.001	0.53	0.69
*SD1*/*SD2*	1.40 (0.29)	−0.16 (0.29)	−0.96 (0.30)	< 0.001	0.05	0.002	0.72	0.03

Values are estimated marginal means of Z scores (SE). *p* values are derived from linear mixed effect models

*Group C* controls (n = 38), *Tot* total, *VLF* very low frequency, *LF* low frequency, *HF* high frequency, *SE* standard error

*p* value < 0.05 is considered statistically significant

A subgroup analysis by Wilcoxon test was carried out on data from patients with multiple Holter-ECG recordings. The same pattern of results was noted: increased RR interval and *SD2*, and reduced *SD1*/*SD2* post-BDG as compared with pre-BDG. A significant reduction of *P*_tot,_ *P*_LF,_ and *P*_HF_ and in Poincaré analysis the *SD1*_,_ and *SD1*/*SD2* as compared with before TCPC was also confirmed. Reduction of *P*_VLF_, *SD2* and the ratio *P*_LF_/*P*_HF_ was not statistically significant (data not shown). A subset analysis of patients with HLHS confirmed these results.

No significant differences in HRV were found between the 10 patients who received a heart transplant or died during follow-up compared the remaining patients in the study cohort.

## Discussion

The main findings of this study were that a significant reduction in heart rate, increased *RR* interval and HRV (*SD2*) occurred in children with univentricular heart defects after BDG. Further reduction in all HRV spectral indices one year after complete TCPC surgery, was also seen when compared to pre-TCPC HRV results.

Early diagnosis of arrhythmia is desirable in patients with Fontan circulation as arrhythmia is one of the main causes of postoperative morbidity [18, 19]. Known risk factors for arrhythmia in Fontan patients include older age at time of surgery, duration of follow-up, and worse NYHA class symptoms [19]. In addition to these factors, autonomic nervous control has been suggested to play an important role in the development of cardiac arrhythmias [12]. HRV is a non-invasive method used to study the autonomous control of heart rate. Surgery-related damage to autonomic nerves, ganglia, or to blood vessels supporting these structures could explain impaired cardiac autonomous nervous activity in Fontan patients. In this study mean heart rate after BDG was reduced in 30%, which is a high amount compared to only 6% in a study by Cohen et al. [20]. However, the amount of bradycardia did not increase further after TCPC. SND after TCPC has earlier been reported in a range from 30 to 45% [6, 7]. The reason for the frequent occurrence of SND in patients with Fontan circulation has been discussed frequently. It is considered to be either due to damage to the sinus node during surgery or reduced blood supply to the sinus node that may result in fibrosis [6, 21].

Although results showing reduction in HRV pre-BDG could be explained by pre-existing cardiac autonomic dysfunction in these patients [22], another possibility is that reduced HRV is related to the volume overload of the single ventricle pre-BDG. In adults, abnormal HRV parameters have been found to be significantly and independently associated with congestive heart failure [23]. In children with atrial septal defects, HRV was reduced before, but normalized after transcatheter closure [24]. Thus, volume overload might explain the low HRV seen in patients before BDG procedure.

Post-BDG surgery, *RR* intervals were significantly increased and, consequently, heart rate reduced. Interestingly, pre-BDG heart rate was within normal range (Table 2) in patients compared to controls, suggesting that the reduction in heart rate post-BDG is not related to release of ventricular volume overload alone. Histological studies of the normal human heart, both in adults and children, have shown large populations of ganglia located on the posterior surfaces of the SVC near the junction of the right atrium [16, 25]. Additionally, a study on normal fetal hearts showed dense population of epicardiac ganglia distributed on the posterior-superior surface of the right atrium and between the entrances of both caval veins [26]. It is thus possible that these ganglia are affected during the BDG procedure when the SVC is detached at the entrance to the heart and the cardiac end oversewn. This could explain the marked increase in RR intervals, and also explain why HRV remains low, compared to controls, even after BDG releasing ventricular of volume overload.

In line with previous studies, we found that heart rate was lower (increased *RR* interval) post TCPC surgery when compared with healthy controls [14, 27]. The HRV analysis showed a further reduction in HRV post-TCPC, with significantly lower *P*_HF_ and *SD1* after the TCPC procedure compared to before the TCPC, indicating a reduced parasympathetic action of cardiac autonomic innervation. This is of special interest since the risk for arrhythmia is significantly increased when the autonomic innervation of the heart is sympathetically dominated [28]. The resulting HRV parameters indicating a lower parasympathetic tone in the post-TCPC patients compared to post-BDG patients strengthens and supports findings by Madan et al. [15]. This study compared 15 min recordings of HRV in two groups of patients; patients with BDG and preserved pulmonary blood flow and patients with TCPC. The study found a higher root mean square of successive *RR* interval difference (*RMSSD*) and a lower *P*_LF_ in the BDG group [15].

Previous studies have shown lower HRV in early, post-surgery follow-up assessment verses late, post-surgery follow-up assessment of cardiopulmonary bypass patients [15, 29]. In order to avoid this transient effect in our study, we chose a longer time interval and TCPC surgery (Table 1) before collecting Holter-ECG recordings for HRV analysis.

Abnormal HRV parameters have been found to be associated with congestive heart failure [23]. In a previous study on patients with complete Fontan circulation, no relation between poor ventricular function and HRV could be established [30]. In our study, assessment with echocardiography showed good ventricular function and small AV-insufficiency in most patients. Thus, it is not likely that heart failure alone explains the reduction of HRV after Fontan surgery.

In this study most patients (96%) were operated with the extracardiac conduit variant of TCPC surgery. During this surgical procedure the IVC is transected, and the parasympathetic ganglia located close to the IVC–atrial junction, or at the medial and posterior surface of the IVC, may be affected [16, 25]. We found significant reductions in HRV in our study which supports an earlier study, which reported, reduced HRV in patients with TCPC, not only after surgery to create a lateral tunnel, but also after surgery to create an extracardiac conduit [14]. This indicates that the extracardiac conduit variant may also affect the cardiac autonomic innervation.

### Strengths and Limitations

This study, to the best of our knowledge, is the largest longitudinal study of HRV during the stepwise progression to completion of Fontan circulation. Additionally, all but one Holter-ECG recordings were made without concurrent treatment with beta-blockers or other drugs known to interfere with HRV [17].

## Limitations

A lack of data regarding HRV before neonatal surgery. This would have been of interest since most patients (96%) had neonatal cardiac surgery, which may affect HRV. Additionally, 24 h Holter-ECG recordings after each surgical step were not available for each patient.

## Conclusions

This study shows markedly reduced heart rate (increased *RR*-interval) post BDG surgery and further significant reductions in HRV after completion of TCPC. These findings add to the current knowledge regarding autonomic regulation of cardiac rhythm and offer a possible explanation for postoperative arrhythmic events in patients with univentricular heart defects undergoing conversion to Fontan circulation.
